# The biology of uveal melanoma

**DOI:** 10.1007/s10555-017-9663-3

**Published:** 2017-02-22

**Authors:** Adriana Amaro, Rosaria Gangemi, Francesca Piaggio, Giovanna Angelini, Gaia Barisione, Silvano Ferrini, Ulrich Pfeffer

**Affiliations:** 1 Laboratory of Molecular Pathology, Department of Integrated Oncology Therapies, IRCCS AOU San Martino – IST Istituto Nazionale per la Ricerca sul Cancro, L.go Rosanna Benzi 10, 16132 Genoa, Italy; 2 Laboratory of Biotherapies, Department of Integrated Oncology Therapies, IRCCS AOU San Martino – IST Istituto Nazionale per la Ricerca sul Cancro, Genoa, Italy

**Keywords:** G-protein signaling, YAP/TAZ signaling, Immune checkpoint blockers, Targeted therapy, Molecular classification

## Abstract

Uveal melanoma (UM), a rare cancer of the eye, is distinct from cutaneous melanoma by its etiology, the mutation frequency and profile, and its clinical behavior including resistance to targeted therapy and immune checkpoint blockers. Primary disease is efficiently controlled by surgery or radiation therapy, but about half of UMs develop distant metastasis mostly to the liver. Survival of patients with metastasis is below 1 year and has not improved in decades. Recent years have brought a deep understanding of UM biology characterized by initiating mutations in the G proteins GNAQ and GNA11. Cytogenetic alterations, in particular monosomy of chromosome 3 and amplification of the long arm of chromosome 8, and mutation of the BRCA1-associated protein 1, BAP1, a tumor suppressor gene, or the splicing factor SF3B1 determine UM metastasis. Cytogenetic and molecular profiling allow for a very precise prognostication that is still not matched by efficacious adjuvant therapies. G protein signaling has been shown to activate the YAP/TAZ pathway independent of HIPPO, and conventional signaling via the mitogen-activated kinase pathway probably also contributes to UM development and progression. Several lines of evidence indicate that inflammation and macrophages play a pro-tumor role in UM and in its hepatic metastases. UM cells benefit from the immune privilege in the eye and may adopt several mechanisms involved in this privilege for tumor escape that act even after leaving the niche. Here, we review the current knowledge of the biology of UM and discuss recent approaches to UM treatment.

## Introduction

Uveal melanoma (UM) is a rare disease but the most frequent non-cutaneous melanoma and the most frequent primary cancer of the eye in the adult. In recent years, our understanding of this disease has made a leap forward through the identification of the molecular players likely responsible for tumor initiation and progression. The process of multistep carcinogenesis is now known in considerable detail, perhaps better than for any other neoplasia, and prognosis can be made with utmost precision. This is contrasted by the lack of adjuvant therapy and low efficacy of therapy for metastatic UM, leading to survival rates that have not significantly changed over decades.

This review gives a general overview of the current knowledge in the field of UM, incorporating the most relevant findings on the biology of this disease and their implications in clinical management. Reference to recent reviews that give more detailed descriptions is given wherever possible.

## Clinical features of uveal melanoma

### Epidemiology

Approximately 5% of all melanomas affect the eye, making it the most common site for melanoma development after the skin [1]. The vast majority (85%) of ocular melanomas occur in the uveal tract, which is the vascular layer of the eye (comprising the choroid, the ciliary body, and the iris), and hence are known as UM. Conjunctival melanoma is a rare tumor that develops in the mucous membrane lining the inner surface of the eyelids and the forepart of the eyeball. The clinical and histopathological features of conjunctival and uveal melanomas are clearly different; hence, the two entities should not be confused. Uveal melanoma has molecular affinities with melanocytic tumors of the central nervous system [2] whereas conjunctival melanomas show mutation patterns similar to cutaneous melanoma (CM) [3, 4].

The incidence of UM in the USA is 4.3 per million (4.1–4.5; 95% confidence interval [CI]) with a prevalence in males (males, 4.9 [4.6–5.2], 95% CI; females, 3.7 [3.5–3.9], 95% CI). Of the cases registered, 97.8% occurred in the white population [5]. There is a strong difference in the incidence for different ethnic groups: the annual age-adjusted incidence is 0.31 for Afro-Americans, 0.38 for Asians, 1.67 for Hispanics, and 6.02 for non-Hispanic whites [6], yet prognosis does not differ for ethnic groups [7]. The European Cancer Registry-based study on survival and care of cancer patients (EUROCARE) for the years 1983–1994 reported similar incidence rates with a characteristic increase from south to north, from <2 per million in Spain and Southern Italy to >8 per million in Norway and Denmark [8]. This is consistent with increasing incidence observed with increasing latitude in the USA (4.91-fold from 20–22° to 47–48°) [9].

UM has been reported in patients of all ages, but only 1% of cases occur in younger patients under the age of 18. The incidence increases with age, peaking at the age of 70. The age-adjusted incidence rate was stable between 1973 and 1997 in the USA [5]. More recently, an increase in the mean age at diagnosis for the interval between 1973 and 2009 was described based on the analysis of 7043 UM patients from the Surveillance, Epidemiology, and End Results Program (SEER) database [10]. This increase is most likely attributable to the growing life-span and more thorough screening for eye diseases.

Five-year relative survival of UM patients was 68.9% [11], 81.6% [12], and 81.4% [13] for cases diagnosed in Europe (1983–1994), the USA (1973–2008), and Western Australia (1981–2005), respectively. Death rates were slightly elevated in older patients and in males. The general trend in prolonged survival after cancer diagnosis [14, 15] does not apply to UM: no significant differences in overall survival or disease-specific survival were observed between 1973 and 2009 when the data were stratified by year of diagnosis [10–12]. UM thus belongs to the few cancers with stable or increasing death rates.

### Etiology

The etiology of UM is still unclear. UV radiation has clearly been identified as the major risk factor for CM [16], but the role of UV radiation in the development of UM is still under debate [17]. Cornea, lens, and vitreous body absorb almost all wavelengths below 300 nm and much of the spectrum between 300 and 400 nm [18]. However, age-dependent alterations of the vitreous body [19] might alter the absorptive capacity of the latter. The associations between UM risk and blue iris or a generally weakly pigmented phenotype [20, 21] and sun exposure [22] suggest a role for UV radiation in the etiology of UM. A meta-analysis of 133 reports on UV-associated risk factors for UM showed a significant correlation for welding (OR = 2.05; CI 1.20–3.51) but not for outdoor leisure activities (OR = 0.86; CI 0.71–1.04), occupational sunlight exposure (OR = 1.37; CI 0.96–1.96), and latitude of birth (OR = 1.08, CI 0.67–1.74) [23]. If there is a role for UV light in UM etiology, it is certainly by far weaker than that for CM. The etiologic effect of UV radiation for UM is likely too weak to overcome confounding factors such as co-distribution of weakly pigmented skin and iris and latitude, co-occurrence of UV radiation with light of longer wavelengths, and protective, vitamin D-mediated effects of sun exposure [24]. Violet/blue light, the most energetic form next to UV light, has also been cited as a potential risk factor for UM [25]. Genetic variants on chromosome 15q13.1, close to the genes HERC2 and OCA2 on 15q12 that are involved in the determination of eye color, have been found associated with UM risk [26], and the G proteins GNAQ and GNA11 that are frequently mutated in UM [27, 28] are involved in the determination of skin color in mice [29].

In contrast to inconclusive epidemiological data, molecular data can clearly exclude a typical UV-associated mutational spectrum for UM; in fact, it shows a relatively low mutational load when analyzed by whole exome sequencing and no enrichment for UV-typical C>T transitions at dipyrimidine sites [27, 28, 30–32]. UV-induced mutations in the promoter of the human Telomerase Reverse Transcriptase (TERT) gene occur in approximately 70% of CMs [33, 34] but are rare in UMs [35–37]. Hence, if light has a role in UM carcinogenesis, it certainly acts in a different manner than in CM.

### Diagnosis and treatment

UM can develop without any symptom and is diagnosed by routine ophthalmic examination [38]. It often causes painless distortion of vision and other non-specific visual symptoms [39]. Diagnosis of UM relies primarily on clinical examination and ocular ultrasonography [40]. On the hand of an experienced ocular oncologist, elevated levels of accuracy [41] and first visit detection rates [38, 39] have been documented, minimizing the need for invasive diagnostic biopsy [42]. There might be an effect of the time of intervention especially for smaller tumors, and diagnostic delay in older patients might affect prognosis [43]. Treatment options include local radiotherapy (^106^ruthenium or ^125^iodine brachytherapy, proton beam therapy, or stereotactic radiosurgery) or surgery (local resection, endoresection, or enucleation) [42]. Neoadjuvant phototherapy has been proposed with the scope of reducing side effects of brachytherapy [44].

Local control of disease reaches 96.4% after proton beam therapy [45, 46], but local recurrence can arise up to almost 10 years after primary therapy and determines an increased metastatic risk [47, 48].

### Metastatic disease

Despite successful local treatment, 25 and 34% of UM patients develop metastases within 5 and 10 years, respectively [49]. The long-term cumulative melanoma-related mortality rate 25 years after primary treatment is over 50% for medium and large tumors [50]. High-risk cases (see below) should be integrated in a lifelong surveillance program including liver imaging for early detection of metastases [42] since it is the first site of metastasis of UM in most cases [49, 51, 52]. Based on self-reported outcome measures, quality of life reaches levels of the healthy population 6 months after treatment with some stress for younger and female patients and depression in patients with bad prognosis [53].

At present, there is no approved adjuvant therapy for UM, a fact that is in striking contrast with the elevated precision in prognostication (see below). Interferon-α2a (IFN-α2a [45] and methanol-extracted residue of Bacillus Calmette–Guerin [46] have been tested as an adjuvant therapy but did not affect survival. A trial with 22 patients to test adjuvant intra-arterial hepatic Fotemustine showed effects on survival together with considerable toxicity but did not reach statistical significance [47]. Several adjuvant trials are registered at the clinical trials service of the National Institute of Health (clinicaltrials.gov). The anti-vascular endothelial growth factor (VEGF) drug Avastin is being tested in a neoadjuvant setting for its capacity to reduce larger UM prior to therapy (NCT00596362^1^). Dendritic cells and vaccine therapies are being tested (NCT00929019, NCT01983748, NCT00020475) as well as the anti-receptor tyrosine kinase drugs crizotinib (NCT02223819) and sunitinib alone (NCT02068586) or in combination with tamoxifen and cisplatin (NCT00489944), and the histone deacetylase inhibitor valproic acid (NCT02068586). Adjuvant chemotherapy is presently investigated in two trials (dacarbazine in combination with recombinant IFN-α2b [NCT01100528] and fotemustine [NCT02843386]) as well as prophylactic hepatic irradiation (NCT02336763). All these trials rely on advanced prognostic testing (see below). UM is also contemplated in several trials for CM.

The mean survival after diagnosis of metastatic UM is approximately 1 year [54, 55], but a considerable proportion of patients survive more than 4 years [51]. Resection of liver metastases can be performed in relatively few patients where it shows some advantage in survival [56–59] and appears to work better for UM than for CM [60]. Hepatic intra-arterial chemotherapy improves progression-free survival but not overall survival [61]. A phase II trial reveals a 14-month gain in survival [62], indicating that isolated hepatic perfusion is active on established liver metastases [63, 64]. A phase III trial is presently recruiting [62].

At present, ClinicalTrials.gov lists 69 trials for metastatic UM (for recent reviews, see [65, 66]). Targeted therapies based on the activation of the mitogen-activated protein (MAP) kinase pathway by mutated G protein GNAQ or GNA11 have been tested in several clinical trials. Selumetinib that inhibits the MAP kinase kinase enzymes MEK1 and MEK2 showed a slight improvement in progression-free survival [67] that, according to a preliminary report, was not confirmed in a phase III trial [68, 69]. Selumetinib is also being tested in combination with temozolomide (NCT01143402), and the maximum tolerated dose of intermittent selumetinib is being investigated (NCT02768766) [69]. Bevacizumab was tested in combination with dacarbazine showing modest activity [70]. A pilot study with the kinase inhibitor sunitinib showed a potential clinical benefit that was independent of the expression level of the target kinase, c-Kit [71]. An institutional review from Mayo Clinic showed that local therapies were superior to kinase inhibitors [72].

For now, MAP kinase-targeted therapies for UM cannot equal the successes obtained for CM. Similarly, immune checkpoint blockade therapy using antibodies directed against immunomodulatory receptors and ligands induces long-term survival in a considerable portion of CM patients [73] but shows only limited activity in metastatic UM patients with extended responses in some patients [74–83]. A recent phase I trial with AM0010, a pegylated recombinant IL-10, showed responses of solid tumors with BRCA1-associated protein 1 (BAP1) mutations, among which is a UM [84].

Many preclinical studies have addressed UM therapy by analyzing the expression and function of pathways that are target for existing drugs [85–118]. These studies have not led to clinical trials, or the trials did not confirm the activity of the drug in UM patients, as for example for c-Kit-targeted treatment with Gleevec [87].

Table 1 summarizes these efforts.

**Table 1:** Preclinical studies with molecularly targeted drugs

Target	Drug	Effect	Cellular models	*In vivo*	Reference
EGFR	Cetuximab	Triggers NK cells to antibody-dependent cellular cytotoxicity and TNF-α release	MEL285, MEL290, OCM8, UPMM3		[85]
	Gefitinib	Reduces EGFR phosphorylation	MEL285, MEL290, UPMM3		[85]
	879127-07-8	Decreases proliferation and induces apoptosis	M619, C918, MUM2B, MUM2C, OCM1, OCM3, OCM12, MEL285, MEL202, 92.1, OCM1A, OMM1.3		[86]
	Monoclonal αEGFR antibodies	Inhibits metastases, increases susceptibility to TNF-mediated cytolysis	OCM1, OCM3, OCM8, OM431, 92.1	Yes	[102]
BCL2	siRNA	Enhances apoptosis and cell cycle arrest	OM431		[104]
FGF2/FGFR1	Antisense oligo	Reduces cell proliferation	MEL270, MKTBR, OCM1, SP6.5, 92.1		[105]
c-KIT	Imatinib	Reduces cell viability	MEL270, MUM2B, OCM1, OMM2.3, 92.1, μ2F		[106]
VEGF	siRNA	Reduces tubule formation, inhibits the angiogenesis	OCM1, OMM1, 92.1	Yes	[107]
	Bevacizumab	Suppresses primary UM growth and formation of hepatic micrometastases	MEL270, MEL290	Yes	[108]
	Lenalidomide	Inhibits growth of UM cells, primary tumors and metastases	92.1	Yes	[109]
	Sorafenib	Inhibits growth of UM cells, primary tumors and metastases	92.1	Yes	[109]
IGF1-R	Picropodophyllin	Decreases proliferation and induces apoptosis	M619, C918, MUM2B, MUM2C, OCM1, OCM3, OCM12, MEL285, MEL202, 92.1, OCM1A, OMM1.3		[86]
c-Met	shRNA	Decreases proliferation	M619, C918, MUM2B, MUM2C, OCM1, OCM3, OCM12, MEL285, MEL202, 92.1, OCM1A, OMM1.4		[86]
	SU11274	Inhibits cell proliferation	C918, MUM2C, OCM1, OCM 3, OCM 8, 92.1		[110]
	miR144	Inhibits cell proliferation and invasion	C918, MUM2B, MUM2C, OCM1A		[111]
	miR182	G1 arrest and increased apoptotic activity, inhibits cell migration and invasion	M17, M21, M23, SP6.4		[112]
	miR34a	Inhibits cell proliferation and migration	M17, M21, M23, SP6.5		[113]
	siRNA	Inhibits cell proliferation and migration and invasion	M17, M21, M23, SP6.6		[112]
	MK8033	Inhibits cell migration	MEL202, MEL270, MEL285, MEL290, OCM1, OCM3, OMM2.5		[114]
	Crizotinib	Inhibits cell migration and prevents macrometastasis	MEL285, MEL290, OMM1, OMM1.3, 92.1, C918		[98]
MEK1/2	AZD6244 (MEKi)	MEKi or METi treatment alone reduced cell proliferation and modest induction of apoptosis	MEL202, MEL270, MEL285, MEL290, OCM1, OCM3, OMM2.5		[98]
c-Met + MEK1/2	MK8033, AZD6244	Reduces proliferation	MEL202, MEL270, MEL285, MEL290, OCM1, OCM3, OMM2.5		[98]
MITF	miR137	Reduces proliferation	M17, M21, M23, SP6.5		[117]
HSP90	17-AAG	Downregulation of FAK expression	MKTBR, OCM1, SP6.5, UW1, 92.1		[88]
SDCBP1	siRNA	Inhibits cell migration	MEL270, OMM1, OMM2, 92.1		[89]
HDAC	Tenovin-6	Induces apoptosis, suppresses migration, and eliminates cancer stem cells in UM cells	MEL270, OMM1, OMM2.3, 92.1		[90]
	Variostat	Inhibits the growth of UM tumors *in vitro* and *in vivo*	MEL202, OCM1A, 92.2	Yes	[115]
ARF6	NAV2729, siRNA	Reduces UM cell proliferation and tumorigenesis	MEL202, 92.2	Yes	[91]
YAP	Verteporfin	Suppresses the growth of UM cells bearing GNAQ or GNA11 mutations	13 primary cell lines		[92]
PKC	TAK733	Antitumor properties (cell proliferation inhibition) in GNAQ- or GNA11-mutated UM cells	MEL20 06-039, MEL06-045, MEL07-070, MEL08-128, MEL09-196		[116]
	AEB071, AHT956	Selectively inhibits the growth of GNAQ- or GNA11-mutated UM cells	OCM1, OCM3, MEL202, MEL290, MEL285, MEL270, OMM1, OMM2.3, OMM2.5		[94]
	AEB071	Decreases Erk1/2 phosphorylation, inhibits NF-κB activity	C918, MEL202, MEL285, OCM1, OCM3, OMM1, 92.1		[95]
PKC + MEK1/2	AEB071, AHT956, PD0325901, MEK162	Synergistically kills GNAQ/GNA11 mutant UM cells by induction of apoptosis	OCM1, OCM3, MEL202, MEL290, MEL285, MEL270, OMM1, OMM2.3, OMM2.5		[94]
GNAQ	GNAQ knockdown	Suppresses PKC and MAPK signaling in UM cells with GNAQ mutations	OCM1, 3, MEL202, MEL290, MEL285, MEL270, OMM1, OMM2.3, OMM2.5		[94]
mTOR + MEK1/2	Selumetinib, AZD8055	Inhibits BRAF and GNAQ mutant tumor cell viability	C918, MEL270, MEL290, OCM1A, OCM3, 92.1		[99]
NF-κB	Zeaxanthin	Inhibits cell viability, migration, and invasion	SP6.5, C917		[100]
	BAY11-7082	Induces cell apoptosis and inhibits the migration of human UM cells	OM431, OCM1, SP6.5, VUP		[118]
NOTCH	siRNA	Enhances apoptosis and cell cycle arrest	VUP, OCM1		[103]

*siRNA* small interfering RNA

The ADP ribosylation factor 6 (ARF6), not to be mistaken for the tumor suppressor gene (TSG) p16/INK4a-ARF, has recently been identified as a major hub for oncogenic signaling in UM [91]. ARF6 responds to the activation of several pathways that have been targeted in preclinical studies, among which are the epidermal growth factor receptor (EGFR) [119], the VEGF receptor [120], and the wingless-type MMTV integration site family of signaling protein (WNT) [121] pathways. In mice, ARF GTPase-activating proteins control c-kit endocytosis [122].

### Histopathology

The diagnosis of UM is generally made by a trained ophthalmologic oncologist, and only few cases require cellular analyses in order to rule out an ocular metastasis of other solid tumors. Histopathology is primarily required for prognostication of UM [123]. Nuclear grade and cell type are linked to prognosis. Tumors dominated by epithelioid cells have a worse prognosis than those with prevalently spindle-like cells. Tumors with a mixed cell type have an intermediate risk [123–125]. The proportion of spindle cell histology is more frequent in younger patients and has decreased over time [10], probably as a consequence of increased age at diagnosis. Accordingly, metastatic UM shows prevalently epithelioid cells although a component of spindle-like cells is always present [126]. Other histopathological features commonly used for assessing the malignant potential of UM are the number of mitotic figures and the presence of extracellular matrix periodic acid–Schiff-positive closed loops.

More recently, immunohistochemistry has been proposed as a useful prognostic tool for the analysis of nuclear expression of the BAP1. The somatic mutation of this gene, resulting in a loss of protein expression, is associated with metastatic risk [127–130] (see below).

## Genetics of uveal melanoma

### Cytogenetics

The most frequent cytogenetic alterations encountered in UM are monosomy of chromosome 3 and amplification of 8q [131, 132], both associated with poor prognosis [133, 134]. The amplifications are conserved in metastases [135]. Amplification of chromosome 6p [132, 136] and losses of 1p are also frequent [137]. Table 2 summarizes the results of studies analyzing large cohorts by microsatellite analysis [140, 141], fluorescence *in situ* hybridization (FISH) [142, 143], multiplex ligand-dependent PCR amplification (MLPA) [139], or single nucleotide polymorphism (SNP) microarrays [143]. These studies confirm the strong association of monosomy of chromosome 3 with death from metastasis as well as with other histopathological factors such as epithelioid cells, closed microvascular loops, ciliary body involvement, large basal tumor diameter, and tumor thickness. The latter two remain to be important histopathological prognostic factors [144, 145]. Also the highly prognostic chromosome 3 monosomy and 8q amplifications occur independent of each other, and principal component analysis of karyotypes identifies four classes with prognostic relevance: (i) disomy 3/disomic 8q, (ii) monosomy 3/disomic 8q, (iii) disomy 3/8q gain, and (iv) monosomy 3/8q gain [146]. Three to seven copies of 8q could be detected [138]. Digital PCR which offers the possibility to detect rare events in heterogeneous tissue samples might further improve the prognostic power of molecular cytogenetics [147]. All chromosomal alterations except amplification of 6p correlate with the largest basal tumor diameter, indicating that they do not occur at the onset of the disease but they are acquired later on [43].

**Table 2:** Chromosomal deletions and amplifications in uveal melanoma (%)

No. of cases	Sample type	Method	Chromosome									Reference
			3		1p	6p	6q		8q	8p		
			Monosomy	Partial monosomy	Losses	Gains	Losses	Gains	Gains	Losses	Gains	
356	Local resection or enucleation	FISH	47						37			[138]
452	Local resection or enucleation	MLPA	61			54	22		63	18		[139]
500	Fine needle aspiration biopsy	MS	25	27								[140]
374	Enucleation	MS	56						54			[141]
220	Enucleation	FISH	61			42	30		61			[142]
320	Fine needle aspiration biopsy or enucleation	SNV arrays	45	6	18	32	17	6	51	18	16	[143]
Total			54			42	22		53			

It is clear that monosomy of chromosome 3 is the single strongest cytogenetic factor to predict UM metastasis. Almost all cancers show chromosomal aberrations. Often, tumors contain a mutated, non-functional allele of a TSG and the functional wild-type allele is lost by deletion. This has also been shown for UM where the tumor suppressor gene BAP1, located on 3p21.1, frequently shows somatic mutations in the only allele present in tumors with monosomy of chromosome 3 [148]. However, in contrast to other tumors, a loss of BAP1 function appears not to be sufficient. Most metastatic tumors show complete monosomy, but there are many cases with partial monosomy, indicating that it is not the result of mitotic non-disjunction of the entire chromosome. Interestingly, smaller tumors with a lower metastatic risk show a higher proportion of cases with partial monosomy 3 [140]. Hence, there must be a selective advantage for the loss of an entire chromosome 3. Principal component analysis of the results of an MLPA-based screening of chromosome 3 confirmed the association of metastasis risk with the loss of the entire chromosome rather than a single region therefrom [146]. The identification of the smallest overlapping regions (LOR) usually leads to the identification of TSGs. However, this approach has not worked out for UM where two LORs were identified but none of them contained BAP1 [149] that was later identified by exome sequencing. A similar, microsatellite-based analysis identified an adjacent lesion in 3p25.1–3p25.2 that also does not contain BAP1 [150]. Several cases with deletions spanning the BAP1 locus were identified in a bacterial artificial chromosome-based comparative genome hybridization study, yet the gene was not identified [151]. Isodisomy of chromosome 3 also occurs [152] and is apparently associated with a metastatic risk of UM [153]. The nature of the apparent selective advantage of monosomy or isodisomy over the simple deletion of the wild-type copy of BAP1 and the reason why BAP1 did not consistently show up in the cytogenetics-based tumor suppressor gene research is unknown. The p53 apoptosis effector (PERP) is expressed at low levels in UM with monosomy 3 [154], and the tumor protein p63, encoded by the TP63 gene, located on chromosome 3q27–29, has been shown to be necessary to promote apoptosis in tumor protein p53 (TP53) wild-type UM cell lines. Yet, the role of monosomy remains unclear since the study used disomic cell lines and did not analyze UM tumors [155].

Copy number alteration analysis using SNP arrays has revealed several amplifications and deletions, among which are amplifications on chromosome 6q25.2 containing the membrane-associated guanylate kinase interacting protein-like 1 (CNKSR3) that was amplified in a specific rare subset of UM with monosomy of chromosome 3 and extended metastasis-free survival [156].

### Somatic mutations

Whole exome sequencing [30, 32, 157], including The Cancer Genome Atlas (TCGA) data (http://cancergenome.nih.gov/), shows that UM has relatively few recurring mutations, indicating a low genetic complexity and the absence of genomic instability. Many studies report mutation frequencies that are summarized in Table 3 [27, 28, 30, 32, 35, 83, 126, 128, 130, 147, 148, 157–172]. Initially, BRAF mutations had also been described for UM [173, 174] but these reports have not been confirmed.

**Table 3:** Frequent somatic mutations in primary uveal melanoma

No. of samples	Potential driver mutations				Metastasis drivers			Reference
	GNAQ	GNA11	CYSLTR2	PLCB4	BAP1	SF3B1	EIF1AX	
67	49% (33/67)							[158]
48	46% (22/48)							[27]
75	53% (40/75)							[159]
22	36% (8/22)							[2]
27	44% (12/27)							[2]
163	48% (55/115)	34% (55/163)						[28]
57	47% (7/15)				47% (27/57)			[148]
91	47% (43/91)	44% (40/91)						[160]
102	42% (36/86)	52% (43/83)			38% (32/85)	19% (19/102)		[32]
117	25% (3/12)	58% (7/12)			58% (7/12)	15% (18/117)	8% (1/12)	[161]
111^a^	41% (9/22)	41% (9/22)			11% (5/45)	20% (20/111)	21% (23/111)	[30]
92	35% (6/17)	43% (40/92)						[162]
74					47% (35/74)			[128]
50	18% (9/50)	20% (10/50)						[156]
46	42% (19/45)	33% (15/46)			32% (12/38)	10% (3/31)	19% (7/37)	[35]
116	46% (52/113)	35% (41/116)			50% (56/111)	10% (11/110)	16% (18/111)	[163]
66	41% (27/66)	50% (33/66)						[147]
123	48% (59/123)	46% (57/123)						[164]
23	35% (8/23)	39% (9/23)		9% (2/23)	35% (8/23)	9% (2/23)	17% (4/23)	[157]
74						22% (16/74)		[165]
7	29% (2/7)	57% (4/7)			57% (4/7)			[130]
81	44% (36/81)	44% (36/81)			45% (29/64)	23% (19/81)	17% (14/81)	[166]
158	52% (67/130)	44% (57/130)			51% (81/158)	22% (29/131)	17% (23/133)	[167]
133	49% (67/132)	38% (44/117)			75% (9/12)	24% (32/133)	21% (28/133)	[168]
15^a^	53% (8/15)	40% (6/15)						[83]
136	43% (58/136)	49% (67/136)	3% (4/136)	4% (5/136)	35% (48/136)	18% (24/136)	13% (18/136)	[169]
65					43% (28/65)			[130]
2013	45% (686/1518)	42% (573/1373)	3% (4/136)	4% (7/159)	43% (381/887)	18% (193/1049)	18% (136/777)	
Range (%)^b^	18–53	20–58	3	4–9	32–75	9–24	8–21	

^a^Selected patients

^b^Studies with selected patients not considered

GNAQ [27] and GNA11 [28], two genes encoding Gα subunits of G proteins, are considered the major drivers of UM carcinogenesis since they are found in a mutually exclusive manner in over 80% of UM (see Table 3) where they occur in most if not all of the cells forming the tumor [158]. A significantly lower frequency has been reported for patients from China [170]. Mutations in GNA11 but not GNAQ have been found more frequently in metastatic UM [28, 126]. All mutations of GNAQ and GNA11 in UM involve the same two hotspots in both genes, Q209 and, less frequently, R183, in the Ras-like GTPase domain [175]. These mutations are also encountered in blue nevi and nevi of Ota [176] and melanocytic tumors of the central nervous system [177]. The somatic mosaic GNAQ R183Q mutation is associated with the neurocutaneous disorder Sturge–Weber syndrome and non-syndromic port wine stains [178]. The exclusively somatic nature of the mutation in the Sturge–Weber syndrome and the absence of GNAQ and GNA11 germline mutations in familiar cases of UM [179] suggest that the germline mutation, if present in all cells, is not compatible with life.

Consistent with their oncogenic function, GNAQ and GNA11 mutations in UM are activating mutations. GNAQ and GNA11 are activated by the serotonin (5-HT) receptors 2A and 2B (HTR2A and HTR2B) [180]. The role of serotonin in uveal melanocytes is unknown, but the involvement of the neurotransmitter in the regulation of proliferation, cell shape, and migration of melanocytes has been described for frog skin [181]. Interestingly, HTR2B, the gene encoding the serotonin receptor 5-HT2B, is overexpressed in UM with high metastatic risk [182, 183] despite the fact that the activating mutations in GNAQ and GNA11 already activate the pathway. The endothelin receptor types A and B (EDNRA and EDNRB) also signal via GNAQ and GNA11 [184, 185]. Endothelin 2 is differentially expressed in UM [186], and its receptor EDNRB is downregulated in metastatic UM [187]. Evidence from mice indicates that endothelin signaling could be specific for Schwann cell precursor-derived melanocytes that also populate the uvea [188].

GNAQ and GNA11 activate the classical G protein signaling cascade via inositol-3-phosphate, diacylglycerol, and cyclic AMP, leading to the stimulation of MAP kinases, protein kinase B (Akt) and protein kinase C (PKC), phosphoinositide 3-kinase (PI3K), and mechanistic target of rapamycin (mTOR) [189]. More recently, GNAQ and GNA11 have been shown to activate the transcription factor complex YAP/TAZ in a HIPPO-independent manner [93, 190]. HIPPO has been identified as an important regulator of organ size, and its involvement in several cancer types has recently been appreciated [191]. At present, it is not clear which of the two pathways, YAP/TAZ or MAP kinase, is more important in UM. Interestingly, activation of YAP/TAZ has been shown to confer resistance to BRAF-targeted therapy in CM [192]. MAPK-targeted therapy has so far not been successful in UM (see above), and YAP/TAZ inhibition might become an alternative. The YAP/TAZ pathway can be interrupted by the photodynamic drug verteporfin [93] and through inhibition of the mevalonate pathway [193]. The stimulation of several signaling pathways including activation and nuclear translocation of β-catenin by mutated GNAQ and GNA11 proteins relies on the small GTPase ARF6 [91]. Its inhibition by the small molecule inhibitor NAV-2729 leads to the reduced growth of GNAQ mutant cells *in vitro* and *in vivo* [91].

Fifteen to twenty percent of UM show no mutations in GNAQ and GNA11 genes. The analysis of whole exome sequencing data in double wild-type UM has led to the identification of a L129G-activating mutation in the cysteinyl leukotriene receptor 2 (CYSLTR2) gene, coding for a G protein-coupled receptor. The mutation leads to constitutive activation of GNAQ signaling [169]. The D630Y mutation in the phospholipase Cβ4 (PLCB4) gene that has been observed in several GNAQ and GNA11 wild-type UM cases activates the same pathway acting downstream of GNAQ by catalyzing the formation of inositol 1,4,5-trisphosphate and diacylglycerol from phosphatidylinositol 4,5-bisphosphate [157]. In only a minor part of UM, no putative oncogenic mutation has been found so far. Mutations in the genes GNAQ, GNA11, CYSLTR2, and PCLB4 are likely the initiating driver mutations of UM.

The BAP1 gene encodes a nuclear ubiquitin carboxy-terminal hydrolase with deubiquitinase activity [194] that behaves like a classical TSG in UM [148]. It is located on chromosome 3p21.1, and 84% of metastatic UM maintain only the mutated allele of BAP1 [148]. Germline mutations in BAP1 are associated with several familiar cancers, among which are lung adenocarcinoma, meningioma, mesothelioma, CM, and UM [195–200]. The specific type of cancer that develops in BAP1 mutation carriers might depend on additional modifier genes [201]. Eight percent of patients with metastatic UM carried germline BAP1 mutations [198], and the penetrance in consideration of all types of malignancy approaches 70% and might increase with increasing age of the unaffected BAP1 carriers [200]. Germline BAP1 mutations could be more frequent in patients with early onset of UM [202]. Similar to what has been observed for other TSGs, loss of function mutations can occur over the whole coding region of the gene and mutation is associated with a loss of protein expression. For prognostication, immunohistochemistry has been established as a reliable method to predict BAP1 mutation [127–130].

BAP1 is associated with BRCA1 but does not lead to its deubiquitylation. Instead, it deubiquitylates BRCA1-associated RING domain 1 (BARD1) and modulates the E3 ligase activity of the complex formed by BRCA1 and BARD1 that is needed for the regulation of the DNA damage response [203]. The tumor suppressor activity of BAP1 requires deubiquitylation activity and nuclear localization [204]. Among the substrates of BAP1, there is the repressive transcriptional regulator host cell factor 1 (HCF1) that acts on E2F-responsive promoters [205]. BAP1 also controls transcription and chromatin structure via histone H2A deubiquitylation [206] and through its interaction with HCF1 and the transcription factor Yin Yang 1 (YY1) affecting the transcription of many genes [207, 208]. Consistent with the profound effect on the transcription profile of the cells, depletion of BAP1 determines cell dedifferentiation and the acquisition of stem cell features [209]. The effect on H2A can be controlled by histone deacetylase inhibitors [115] that are presently tested in the clinics as a single agent (NCT00121225, NCT01587352) or in combination with immune checkpoint blockers (NCT02697630).

The Splicing Factor 3b Subunit 1 gene (SF3B1) is mutated in positions R625 [32, 161] and K666 [165] in a minority of UM cases, almost always in the absence of BAP1 mutations. These mutations only rarely occur in CM [210, 211]. SF3B1 is frequently mutated in hematopoietic malignancies with, however, another mutation hotspot in K700 [165] with likely the same or similar functional consequences. SF3B1 mutation alters its function in RNA splicing, leading to the selection of cryptic splice sites [165, 212, 213] thus affecting many primary transcripts. It is therefore not possible to classify SF3B1 mutations in activating or inactivating mutations. SF3B1 mutations define a subclass of UM with chromosome 3 disomy and without BAP1 mutations that nevertheless develop metastases after extended latency [167, 214, 215]. This subclass is distinguished by overexpression of the preferentially expressed antigen in melanoma (PRAME) [215] which acts as a dominant repressor of retinoic acid receptor signaling [216], and its expression is controlled by promoter methylation and is associated with a metastatic risk in SF3B1 as well as in BAP1 mutated cases [217].

**Fig. 1:**
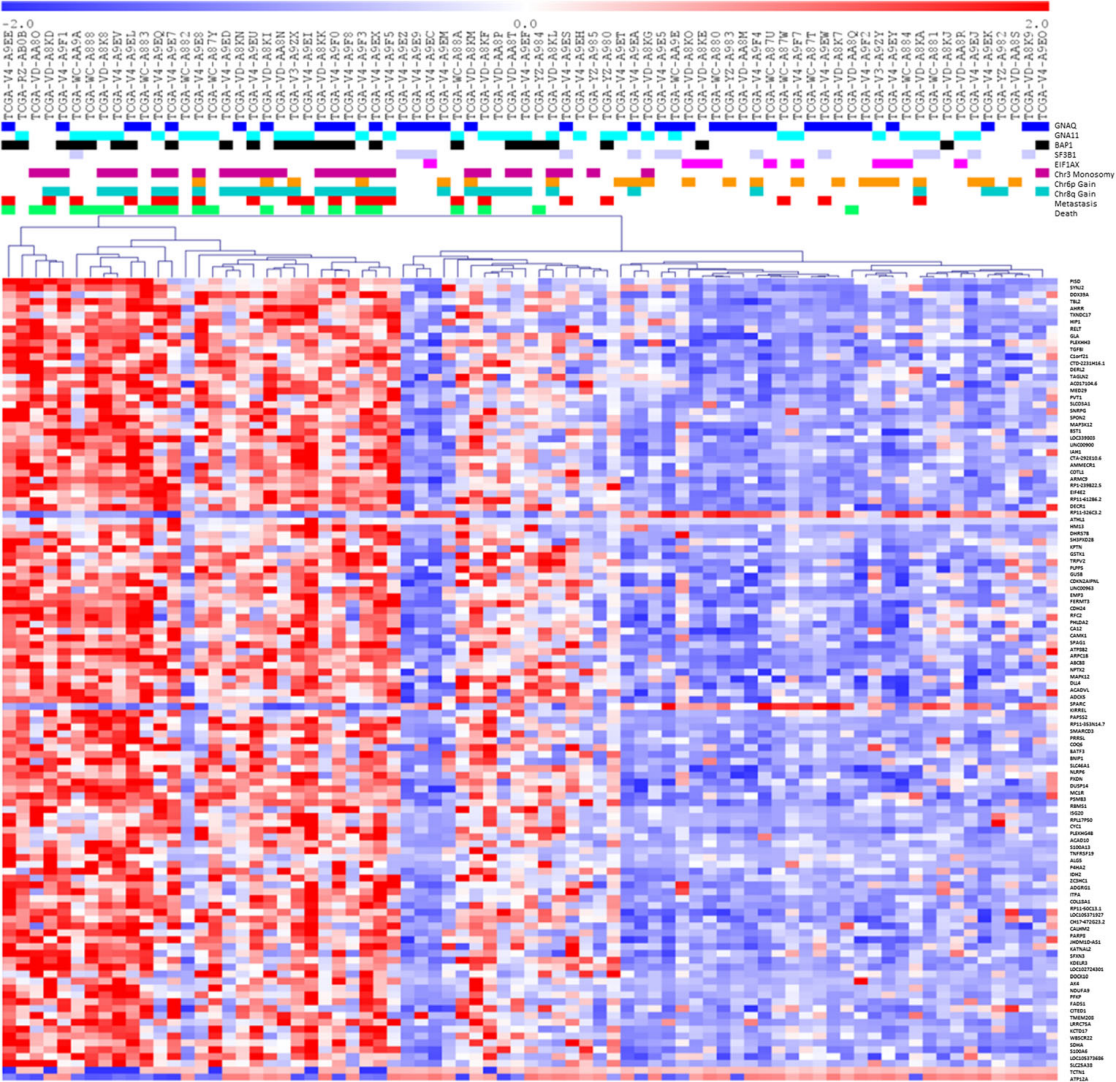
Gene expression and mutational status in UM. Gene expression data from 79 cases of primary UM from the TCGA cohort were used for the identification of genes that are differentially expressed in cases that yielded distant metastases later on as compared to cases without progression to metastatic disease. Statistically significant gene expression differences were obtained by analysis using the bootstrapping algorithm *significance analysis of microarrays* as described [84]. Expression values over the mean value are indicated in *red*, values below the mean in *blue*, and values at the mean in *white*; the intensity of the *color* corresponds to the distances from the mean as indicated in the *red-blue bar on top of the diagram*. Differentially expressed genes were used for hierarchical clustering performed as described [84]. The dendrograms on *top* of the gene expression *panel* show the relationship of single samples and their agglomeration in clusters. The differences between the clusters are indicated by the *length of the branches of the dendrograms*. Above the dendrogram, the mutational status for GNAQ, GNA11, BAP1, SF3B1, and EIF1AX is indicated as well as chromosomal alterations (chromosome 3 monosomy, 6p and 8q gains) and the development of distant metastases and the status of the patients at the end of the follow-up. In all these cases, *colored boxes* indicate the presence and *white boxes* the absence of the alteration. Two main clusters are formed, one of which contains most of the cases that developed metastases. The mutation status of these cases evidences the correlation between chromosome 3 monosomy, BAP1 mutation, metastasis, and death. Note that this cohort contains an unusual case with mutations in both GNAQ and GNA11

Missense mutations in the highly conserved amino-terminal portion of the Eukaryotic Translation Initiation Factor 1A X-Linked (EIF1AX) gene also occur prevalently in the absence of BAP1 mutations in disomic, low-risk UM [30]. The association of EIF1AX mutations with a low-risk profile has been confirmed in an independent study [163]. EIF1AX stimulates the transfer of methionyl initiator tRNA (Met-tRNAi) to the small ribosomal subunit during translation initiation. The functional consequences of these mutations are unknown, and the involvement of alternative start codon recognition has been suggested [30]. BAP1, SF3B1, and EIF1AX mutations are almost mutually exclusive and associated with early (BAP1), late (SF3B1), or no (EIF1AX) metastasis (see Fig. 1). EIF1AX mutations are present in 18% of UM (Table 3), likely due to positive selection during carcinogenesis. UM with mutations in this gene have, however, a low risk of metastasis. Hence, EIF1AX is pro-tumorigenic with no or negative influence on metastasis or the link between its mutations and a specific pattern of metastasis is still unknown.

The Catalogue of Somatic Mutations in Cancer (COSMIC, http://cancer.sanger.ac.uk/cosmic) reports 58 mutations of EIF1AX in 33,058 cancers analyzed and occasional copy number changes, gains, and losses. EIF1AX mutations also occur in papillary [218] and anaplastic [219] thyroid carcinoma and serous ovarian tumors [220]. EIF1AX mutations are missense mutations mainly clustered in the amino-terminal portion of the encoded protein with some frameshift deletions in UM and a splice site mutation hotspot in thyroid cancer [220].

Figure 1 shows a combined view of frequent somatic mutations in UM and their associations with clinical features and gene expression in the TCGA dataset of 79 UM cases.

### Gene expression

Several groups have assembled significant numbers of expression profiles of UM samples [182, 183, 221–223] which, in part, are publicly available. The TCGA dataset also contains gene expression profiles (GEPs) obtained by RNA sequencing. From the very first analysis, it was evident that the major hallmark of high-risk UM, chromosome 3 monosomy, is clearly associated with a specific expression profile [182]. This has been confirmed and extended by several other studies [153, 183, 224, 225] and has led to the development of a multigene prognostic classifier [225–229] (see also Fig. 1) with a prognostic power at least similar to cytogenetics-based procedures such as MLPA [139], eventually combined with histopathological criteria [144]. Public UM gene expression datasets build a domain knowledge that can be mined for many studies such as the expression analysis of targets for existing drugs [85, 222].

The gene expression-based classification of UM shows two very robust classes (Fig. 1) similar to what has been observed for breast cancer where the expression of the estrogen receptor α (ESR1) gene defines two classes with clearly distinguished GEPs [230]. The differences between these classes have been proposed to be determined by their origin, from either luminal or basal cells [231]. Cell lineage is in fact a major determinant of GEPs [232]. When applied to UM, this could indicate that tumors with monosomy 3 are derived from different precursor cells than disomic cases with a different intrinsic propensity to metastasize.

Several studies have performed microRNA (miRNA) expression screenings in UM (for a recent review, see [233]). miRNA expression is associated with chromosome 3 status, gene expression profiling classes and prognosis [234]. Functional studies have been performed for selected miRNAs identified in cell lines or very limited sample collections of UM [233]. There is no conclusive evidence for a functional role of miRNA dysregulation in UM due to the lack of large-scale analyses.

## Multistep carcinogenesis

Our current understanding of how cancer develops is based on three concepts that, to some extent, present a conceptual evolution: (i) the two hits hypothesis postulating that transformation of a normal cell into a cancer cell needs at least two irreversible steps [235, 236], usually mutations in oncogenes and tumor suppressor genes; (ii) the multistep carcinogenesis model that predicts distinct morphological changes during tumor development based on specific molecular lesions [237]; and (iii) the hallmarks of cancer concept that establish distinct processes during cancer evolution: sustaining proliferative signaling, evading growth suppressors, avoiding immune destruction, enabling replicative immortality, genome instability and mutation, resisting cell death, deregulating cellular energetics, tumor-promoting inflammation, inducing angiogenesis, and activating invasion and metastasis [238, 239].

**Fig. 2:**
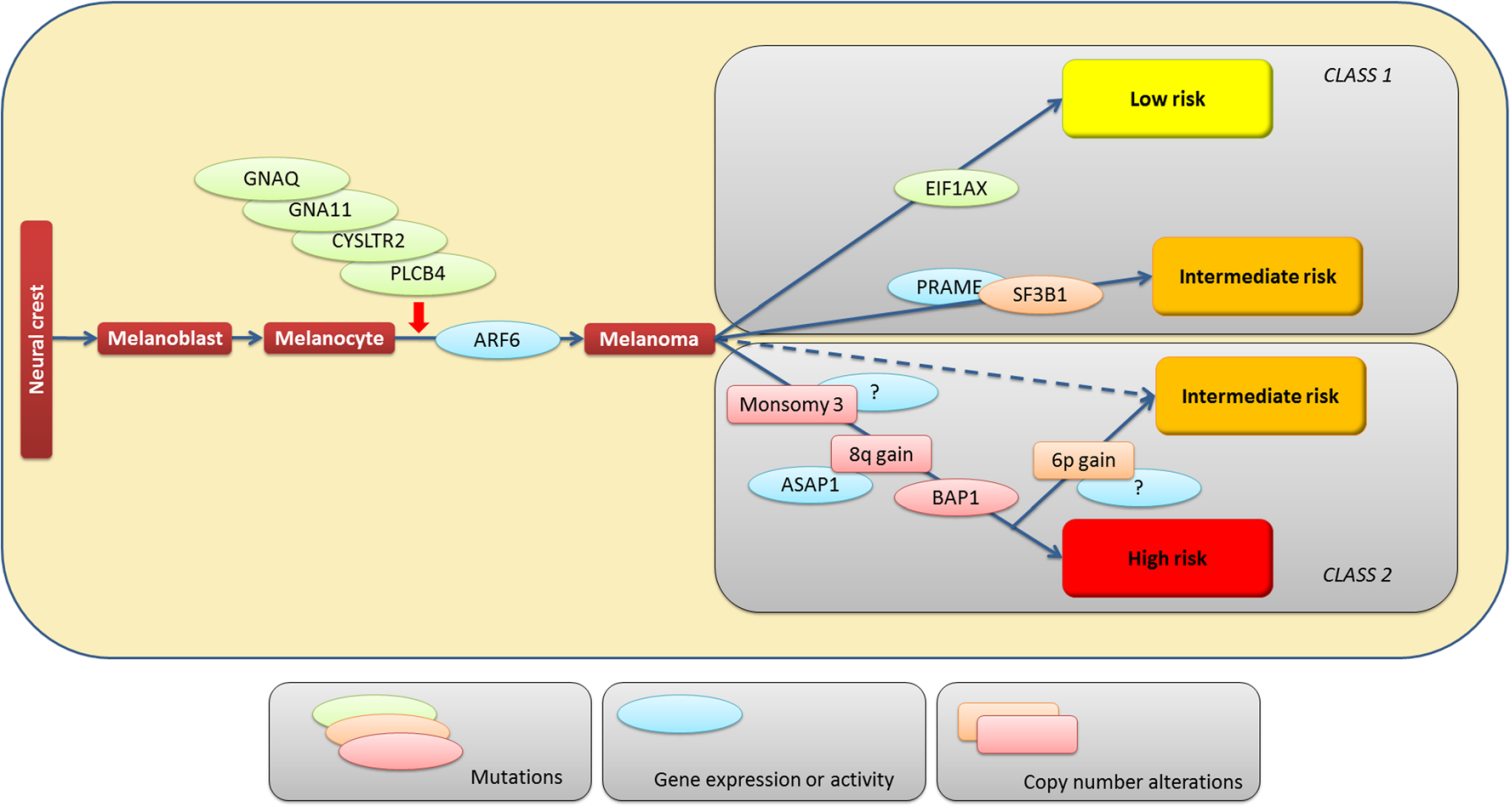
Multistep carcinogenesis of UM. The diagram shows a hypothesis on UM carcinogenesis based on the current state of knowledge of molecular alterations. UM develops from normal melanoblasts or melanocytes and is initiated in most cases by mutations in the Gα proteins, GNAQ or GNA11, or in CYSLTR2 or PLCB4, two genes acting in the same pathway. An important functional mediator of these mutations is ARF6 that is activated. The resulting melanoma can undergo further molecular alterations that influence the risk of progression to metastasis. Loss of one copy of chromosome 3, mutation of the tumor suppressor BAP1, and amplification of chr8q yield a high metastatic risk that can be attenuated by concomitant amplification of chr6p. Intermediate risk is also observed for cases with chromosome 3 disomy in the presence of SF3B1 mutations. Mutations in EIF1AX and the lack of any of these mutations are associated with a low risk of metastasis

A growing body of data describes the accomplishment of the hallmarks by UM and has recently been reviewed in detail [240]. Our current knowledge of the steps of UM carcinogenesis is summarized in Fig. 2. Almost all UMs are characterized by mutations in either GNAQ, GNA11, PLCB4, or CYSLTR2 with few cases showing none of these mutations [27, 28, 157, 169]. These mutations are most likely not sufficient for the establishment of the tumor. The second hit might be an additional mutation or specific systemic condition such as inflammation or angiogenesis. The progression to invasion and metastasis is driven in most cases by the loss of chromosome 3 and mutation of BAP1. Given the fact that partial deletion of chromosome 3 only occasionally yields a metastatic phenotype, additional factors on chromosome 3 are expected. Mutations in SF3B1 can also lead to metastasis in the absence of BAP1 mutations in disomic UM [32].

Amplifications of the long arm of chromosome 8 independently contribute to a metastatic phenotype. The V-Myc Avian Myelocytomatosis Viral Oncogene Homolog (MYC) oncogene is located on 8q, but its expression does not follow the amplification status, instead the gene ArfGAP With SH3 Domain, Ankyrin Repeat and PH Domain 1 (ASAP1) also named DDEF1, AMAP1, or Centaurin-β4, has been proposed to mediate the effects of 8q amplification [241]. This is particularly interesting, given the involvement of ARF6 in UM progression [91] since ASAP1 is a GTPase-activating protein for ARF1 [242] as well as ARF6 [243].

The molecular basis for the apparent protective effect of 6p amplifications [244, 245] is unknown. Chromosome 6p contains genes that are involved in the development of the eye, and congenital deletions lead to eye malformation mainly involving the anterior chamber [246]. Chromosome 6p also harbors the HLA loci and a series of immunomodulatory genes belonging to the B7-CD28 superfamily [247]. HLA loci are not associated with UM risk [248] but might play a role in UM progression [249] through their ability to protect target cells from natural killer cell-mediated lysis [250].

We have recently reported expression of the B7 family member Butyrophilin-Like 2 (BTNL2) gene by UM cells and macrophages that might contribute to the definition of the specific immunologic niche [223].

## Inflammation

In general, chronic inflammation may contribute to early steps of oncogenesis in different tumor types [251]. Indeed, inflammatory cells such as tumor-associated macrophages (TAMs) and several soluble factors are fundamental components of the tumor microenvironment and mediate tumor-promoting effects [252, 253]. The recruitment of monocytes from peripheral circulation and their subsequent differentiation to TAMs is an essential step in establishing the tumor microenvironment, which has several features of a chronic inflammatory state. Several chemokines, cytokines, and protein products of the complement cascade contribute to monocyte recruitment at the tumor site and their differentiation in TAMs [254]. In the microenvironment, TAMs establish multiple cross-talks with cancer cells, endothelial cells, fibroblasts and other mesenchymal cells, and lymphoid cells. These interactions result in tumor progression through the promotion of tumor cell proliferation and survival, angiogenesis, and suppression of anti-tumor immune responses.

Monocytes and macrophages have a highly functional plasticity and may undergo divergent functional polarization in response to activation signals such as cytokines, microbial components, or products deriving from tissue damage or metabolism [255, 256]. In particular, *classical activation* with LPS or IFN-γ induces M1 polarization of macrophages that results in their ability to produce CXCL9 and CXCL10 anti-angiogenic chemokines and pro-inflammatory (e.g., TNF-α) and Th1-polarizing (e.g., IL-12) cytokines. In addition, M1 macrophages acquire cytotoxic properties towards intracellular pathogens and tumor cells. On the other hand, alternative activation with IL-4 or IL-13 induces M2 polarization resulting in the production of CCL17, CCL22, IL-10, pro-angiogenic factors, and matrix metalloproteinases involved in tissue remodeling. However, M1- and M2-type macrophages represent only the two extremes of a broad spectrum of functional states which macrophages can acquire in response to different stimuli. Within the tumor environment stimuli such as hypoxia, cytokines produced by cancer cells (e.g., CCL2, transforming growth factor (TGF)-β, and CSF-1) or by Treg cells (e.g., IL-10), metabolic products, and immune complexes may drive TAM functional polarization mainly towards an M2 status [252].

Several lines of evidence indicate that inflammation and macrophages play a pro-tumor role also in UM [257]. In UM, a high density of macrophages was significantly associated with the presence of epithelioid cells, heavy pigmentation, and elevated density of microvessels. More importantly, the presence of high numbers of TAMs in primary tumors correlated with an increased UM-specific mortality rate at 10 years [258]. Other studies showed that UM TAMs were predominantly CD68+CD163+ M2-type macrophages and that their high density correlated with monosomy 3, ciliary body involvement, increased microvascular density, and worse survival [259, 260]. Monosomy 3 is a well-known predictive marker of a poor prognosis in UM, in relationship to the presence on this chromosome of the tumor suppressor gene BAP1 [148]. It has been proposed that the loss of BAP1 function may favor the generation of an inflammatory tumor environment [261]. Indeed, in a BAP1(+/−) mouse model, intraperitoneal exposure to low-dose asbestos fibers showed higher levels of pro-tumorigenic M2 macrophages and incidence of mesothelioma than in wild-type littermates [262].

TAMs may also contribute to hepatic metastases, where CD68+ TAMs showed more intermediate and dendritic cells associated with a higher microvascular density than the primary UMs. In addition, the survival of metastatic UM patients tended to be shorter if hepatic metastases had a high microvessel density [263]. Altogether, the data from primary or metastatic UM support the hypothesis that TAMs have pro-angiogenic and tumor-promoting activities in human UM. Accordingly, intraocular models of melanoma in syngeneic mice showed a tumor-promoting role of M2 type macrophages, particularly in aged mice [264, 265]. Indeed, depletion of macrophages strikingly inhibited intraocular melanoma growth in a syngeneic model [264] and, more recently, in a transgenic model of UM [266]. Expression of M1/M2 polarization genes is strongly associated with metastatic risk [223].

Regarding the mechanisms stimulating macrophage recruitment, expression of endothelial monocyte-activating polypeptide (EMAP)-II, detected by immunohistochemistry, correlated with the accumulation of macrophages in primary UM. In areas with high EMAP-II, intercellular adhesion molecule (ICAM)-1 was strongly expressed on endothelial cells. These results suggested that macrophage recruitment is facilitated by the chemotactic activity of EMAP-II and by the expression of ICAM-1 on vascular endothelial cells [267]. The vitreal concentration of many pro-inflammatory cytokines and chemokines, such as IL-6, CXCL-8, CXCL-10, CCL-2, CCL-3, CCL-4, CCL-5, and TNF-α, increased in the eyes of UM patients relative to controls. On the opposite, the levels of the anti-inflammatory IL-1R antagonist were lower in UM vitreous. Notably, IL-6 levels positively correlated with high macrophage infiltration [268]. It should be considered that several of these cytokines may exert immune-suppressive and M2-polarizing effects. In addition, co-culture of UM cells with activated T cells upregulated the expression of chemokines and cytokines such as CXCL8, CXCL9, CXCL10, CXCL11, CCL2, CCL5, VEGF, and granulocyte–macrophage colony-stimulating factor (GM-CSF), which coincided with an enhanced ability to stimulate monocyte chemotaxis [269]. These data suggested that the cross-talk between tumor cells and T cells may result in the recruitment of pro-tumoral macrophages. CXCL-8 may be relevant not only as a pro-inflammatory but also as a pro-angiogenic factor. *In vitro* studies suggested that UM may activate IL-8 signaling as an alternative pro-angiogenic pathway [270].

Following local treatment of UM by brachytherapy with ruthenium plaques [271, 272], proton beam irradiation [273], or trans-pupillary thermotherapy [274], intratumoral macrophages are always present or even increased in the treated UM area. These TAMs are frequently engulfed with pigment released by dying tumor cells, thus having the appearance of *melanophages*. Also, macrophages are present in extratumoral tissues since brachytherapy may alter their migration and increase their numbers in the sclera and episclera [272]. TAMs infiltrating tumors treated by trans-pupillary thermotherapy predominantly showed a CD68+CD163+ M2 phenotype [274]. However, eyes enucleated after brachytherapy showed a lower density of microvessels than those from untreated UM [263]. In addition, different from untreated UM, no correlation between the amounts of TAMs and microvascular density was observed in the treated eyes. Altogether, these data suggest that M2-type macrophages are present in UM eyes after local treatments, which leads to tumor cell destruction. Under these circumstances, TAMs may mainly mediate a clearance of damaged UM cells and tissue repair but do not exert pro-angiogenic activity.

A small group of patients showed rapid UM regression associated with inflammation and uveitis following I-125 brachytherapy. Interestingly, these patients had a class 1 tumor, based on gene expression analysis. Class 1 tumors show higher expression of melanocyte differentiation antigens, which are recognized by the immune system, relative to class 2 tumors. Therefore, class 1 tumors may be more immunogenic than class 2 tumors upon antigen release induced by I-125 brachytherapy [275].

## Role of immunity in UM biology and therapy

A growing body of evidence supports an important role of the immune system in the surveillance of cancers. Indeed, in recent years, immunotherapy by immune checkpoint blockers has shown unprecedented long-lasting responses in different metastatic cancers [276]. Cytotoxic T lymphocytes (CTLs), which recognize tumor-associated antigen (TAA) epitopes in an HLA class I-restricted manner, play a major role in tumor cell elimination. Indeed, in different tumors, the presence of an abundant T cell lymphoid infiltrate rich in CD8+ T cells frequently correlates with a good prognosis [277–279].

However, it became clear that tumors evade from immune surveillance through several mechanisms. The *immune editing* or “three e” theory proposes that the immune system efficiently controls tumor growth in a first phase of tumor cell *elimination* [280]. In a second phase of *equilibrium*, the immune system still eliminates immunogenic tumor cells but may also select less immunogenic cell variants. This immune selection process sculpts the features of clinically evident tumors that are characterized by uncontrolled growth due to immune *escape* mechanisms, which are particularly active in the tumor microenvironment.

Among these mechanisms, the *adaptive immune resistance* is due to the induction of programmed death ligand 1 (PDL-1) expression on tumor cells through IFN-γ released by tumor-infiltrating CTLs [276]. In turn, PDL-1 switches off the response of CD8+ CTLs by engaging the PD-1 inhibitory receptor at the T cell surface [281]. Antibodies blocking the PDL-1/PD-1 immune checkpoint restore the silenced immune response at the tumor site, leading to CTL-mediated tumor cell lysis. As a matter of fact, anti-PD-1 or anti-PDL-1 blocking monoclonal antibodies (mAbs) mediate potent and long-lasting anti-tumor responses in metastatic cutaneous melanoma [282, 283] and other metastatic cancers.

The efficacy of immune checkpoint blockers is related to a high load of tumor non-synonymous mutations, which potentially lead to the generation of immunogenic neo-antigens, recognized by the T cells [284]. In cutaneous melanoma, the response to anti-PD-1 therapy has been correlated with the expression of PL-1 on tumor cells, which is geographically associated with infiltrating immune cells [285]. More recent observations related the responsiveness to anti-PD1 mAb with the abundance of tumor-infiltrating PD-1high/CTLA-4high CD8+ T cells, which may correspond to exhausted anti-melanoma-specific CTL [286]. Another clinically relevant immune checkpoint in cutaneous melanoma is CTLA-4, a high-affinity receptor for the B7.1 and B7.2 co-stimulatory ligands. Engagement of CTLA-4 with these ligands prevents their interaction with the co-stimulatory receptor CD28 and switches off T cell activation [287]. The anti-CTLA-4 mAb ipilimumab has been the first immune checkpoint blocker approved for immunotherapy of metastatic melanoma [288].

In spite of their common origin from the neural crests and the shared expression of differentiation antigens (e.g., MART-1, gp100, and tyrosinase), cutaneous melanoma is highly immunogenic, whereas UM is considered as a poorly immunogenic tumor [289–291]. The low immunogenicity of UM may reflect its peculiar site of origin and also its biological characteristics.

It is well known that the eye is an immune-privileged organ and has the ability to prevent the potential damage caused by the immune response through several mechanisms (reviewed in [292]). Indeed, skin allografts implanted in the ocular anterior chamber of rabbits showed prolonged survival, leading to the concept of the eye immune privilege. Also, the injection of foreign antigen into the anterior chamber activates suppressor T cells, which inhibit the systemic immune response against the same antigen, a phenomenon defined as anterior chamber-associated immune deviation (ACAID) [293]. In the mouse, this phenomenon is related to F480+ macrophages, which develop tolerogenic functions in the eye environment. Upon migration in lymphoid organs, these macrophages act as antigen-presenting cells and initiate a response of suppressor T cells [294]. The aqueous humor contains several immune-suppressive factors including, for example, the neuropeptides α-melanocyte-stimulating hormone (MSH) and vasointestinal polypeptide (VIP), the cytokines macrophage migration-inhibitory factor (MIF) and TGF-β2. TGF-β inhibits the pro-inflammatory activity of macrophages and mediates their conversion to tolerogenic APC-mediating ACAID [295]. In addition, TGF-β suppresses T cell responses and mediates the conversion of T cells to immune-suppressive regulatory T cells [291]. Also, the enzyme indoleamine 2,3-dioxygenase (IDO) is expressed in the cells lining the anterior chamber cells and may contribute to inhibiting T and NK cell responses through tryptophan depletion and generation of kynurenines [296, 297].

It has been proposed that UM cells benefit from the immune privilege in the eye and may adopt several mechanisms involved in this privilege for tumor escape, also during the metastatic process [289]. Indeed, UM cells express several immune-suppressive cytokines found in the eye, including, for example, TGF-β and MIF, which are inhibitors of NK cell functions and may therefore contribute to UM metastasis development [298]. In addition, UM cells may also express HLA-E, an MHC class Ib molecule, which binds to the inhibitory receptor NKG2A and blocks NK cell functions [299]. Also, classical HLA class I molecules (A, B, and C) protect target cells from NK-mediated lysis, through the engagement of inhibitory receptors termed KIRs [300]. Interestingly, the loss of classical HLA class I molecules is associated with a better prognosis in UM, opposite to what found in most other tumors. This finding suggested that NK cells are involved in immune surveillance of UM, possibly by reducing the chance of the hematogenous dissemination [250]. Therefore, mechanisms impairing NK cell responses may be relevant in tumor escape during the metastatic progression of UM. Indeed, MIC-A/B class I-like molecules, which are ligands of the NK-activating receptor NKG2D, were found on 50% of primary UM, but not on metastatic lesions [301].

Notably, opposite to findings reported in other tumors, a high density of infiltrating lymphocytes in primary UM has been associated with a worse prognosis [302, 303]. An immunohistochemistry analysis of tumor-infiltrating lymphocytes (TILs) showed that CD8+ CTLs and, to a lesser extent, CD4+ T lymphocytes and Treg cells were present in UM inflammatory infiltrates. In this study, the presence of elevated numbers of TILs correlated with infiltrating macrophages and with chromosome 3 monosomy, which is associated with a worse prognosis [290]. Another study showed that the numbers of CD3+ T cells and CD11+ macrophages were correlated with HLA class I and class II expression, suggesting that low levels of HLA expression may lead to a lower inflammatory infiltrate [304]. Overall, these data indicate that the presence of an inflammatory microenvironment, rich in M2-type macrophages, T cells, and pro-inflammatory cytokines, may favor angiogenesis and UM progression [305].

CD4+CD25+FoxP3+ Treg cells are capable of suppressing Th1/CTL responses and represent a major mechanism of tumor escape in several cancers. Treg cells are present in a fraction of UM accounting for 12 or 24% of primary UM, in two different studies [306, 307]. In another study, UM-infiltrating Treg cells were associated with COX-2 expression and with worse survival. Indeed, prostaglandin E(2) (PGE(2) produced by COX-2 is an inducer of Treg cell differentiation from precursors [307].

Besides M2-type macrophages and Treg cells, it has been proposed that also PDL-1 expression by UM cells may contribute to immune suppression. UM cell lines treated with IFN-γ express PDL-1 [308, 309]. In addition, metastatic and primary UM cell lines upregulating PDL-1 expression upon IFN-γ stimulation suppressed IL-2 production by Jurkat T cells *in vitro*. Either anti-PDL-1 or anti-PD-1 antibodies restored IL-2 production. Although these data indicate that expression of PDL-1 by UM cells inhibits T function *in vitro*, immunohistochemistry showed no PDL-1 expression in primary UM *in vivo* [310]. Similarly, IDO is not expressed in primary or metastatic UM, but it is inducible in UM cell lines by *in vitro* stimulation with IFN-γ [311]. These findings suggest that suppressive mechanisms other than PDL-1 and IDO may dominate in the microenvironment of primary UM.

The weak immunogenic potential of UM may relate to the lower incidence of somatic mutations than those found in sun-exposed skin melanomas [312]. Therefore, sun-shielded UMs may have fewer immunogenic neoepitopes encoded by mutated genes than those found in cutaneous melanoma.

Perhaps the low mutational load may explain the low response rates observed in UM patients treated with immune checkpoint-blocking mAbs. For example, a recent study on 58 patients treated in different academic institutions with anti-PD-1 (pembrolizumab or nivolumab) or anti-PDL-1 mAb (atezolizumab) reported a 3.6% overall response rate, 9% of disease stabilizations, and a progression-free survival of 2.5 months [83]. Also, a phase II study of the anti-CTLA-4 mAb ipilimumab at 3 mg/kg dose has shown limited clinical activity in pretreated metastatic UM [75]. Another phase II study of ipilimumab at 10 mg/kg in treatment-naïve metastatic UM reported better results (10% response rate and overall survival of 10 months) [261].

A further phase II study of the anti-PD-1 mAb pembrolizumab is ongoing (NCT02359851), and particular interest may deserve two clinical studies of the combination of the anti-PD-1 mAb nivolumab with the anti-CTLA-4 mAb ipilimumab (NCT01585194 and NCT02626962). This combination has previously shown improved effects, relative to each mAb alone, in metastatic cutaneous melanoma [313]. Interestingly, also patients with scarce lymphoid infiltrates and low PDL-1 expression in their tumors, a situation commonly found in UM, showed clinical benefit from this combination therapy.

Therefore, although most biological and clinical data suggest that UM is a poorly immunogenic tumor, some piece of evidence suggests that this may not always be the case. For example, a pilot study of a gp100 and tyrosinase-based dendritic cell (DC) vaccination in 14 metastatic UM patients showed a median overall survival of 19.2 months, longer than that reported in the literature [314]. In addition, recent data support the existence of an immunogenic UM subset, accounting for 46% of metastasizing UM, characterized by the absence of melanin pigmentation in the metastases, as detected by clinical MRI. These tumors showed the presence of tumor-infiltrating lymphocytes capable of mounting an efficient anti-melanoma response *in vitro*, suggesting that this subtype of metastatic UM may represent an optimal target for immunotherapy [315].

## Biomarkers

Although less than 1% of UM patients have clinical evidence of metastasis at the time of diagnosis, about half of them bear a lifetime risk of fatal progression. Prediction of metastatic risk in UM patients may be assessed by tumor genotyping to improve the accuracy of clinical and pathological staging [166, 227]. It remains questionable if surveillance strategies for metastases development should differentially apply to patients in accordance with their risk classification after treatment of the primary tumor.

Also, it should be considered that primary UM tissue is not always available for risk assessment and no effective adjuvant therapies have been established so far. Therefore, the detection of subclinical micrometastases before they progress may allow the early recruitment of patients in clinical trials. Indeed, local therapy of a limited number of liver lesions was shown to improve survival [57, 316]. In this respect, the identification of sensitive blood biomarkers would be particularly useful both for the early detection of metastases and to monitor the response to new therapies. Since UM metastatic spread is preponderant to the liver, liver function tests (LFTs), which are quite inexpensive and commonly used in the clinic setting, are mostly used for surveillance. A Finnish study showed a low sensitivity but high specificity of individual LFTs, with lactate dehydrogenase (LDH) being the most sensitive parameter. Notably, the authors showed that the association of LFTs with abdominal ultrasonography (USG) every 6 months would identify over 95% of asymptomatic patients [317]. However, a more recent study showed that the sensitivity and metastasis prediction value of LFTs is very poor, although normal LFTs have a negative predictive value of 90% and identify patients with low chance of having metastases [318]. Also, a retrospective study on a cohort of 263 UM patients showed that only 13% of patients with USG-detectable metastasis had simultaneous abnormal LFTs [319].

In the search for better serum biomarkers, S100-β, a reliable biomarker for monitoring CM, was tested in UM patients but showed limited utility. Indeed, S100-β serum levels showed no correlations with other prognostic parameters or development of metastases [320]. Another protein biomarker considered has been melanoma inhibitory activity (MIA). However, serum levels of MIA increased only after clinical detection of metastases [321, 322]. In another study, the combination of S100-β and LDH performed better in identifying UM metastases than γ-glutamyl transpeptidase and MIA alone [323]. Elevated serum levels of MIA, S100-β, and osteopontin (OPN) correlated with liver UM metastases, and the combination of these three biomarkers enhanced the sensitivity and specificity in detecting hepatic metastases [324]. Serum levels of cytokeratin 18 (TPS) [325], insulin-like growth factor 1 (IGF1) [326], growth differentiation factor 15 (GDF15) [327], and Parkinson protein N7 (PARK7 or DJ-1) [328] are significantly more elevated in metastatic than in metastasis-free patients. An increase in VEGF on serial measurements might indicate the presence of metastases, but its value was limited by the large variability among patients [329]. We recently showed that patients with metastatic UM display significantly higher serum levels of a soluble form of the hepatocyte growth factor receptor c-Met (sc-Met) than metastasis-free patients and healthy donors. Analyses of receiver operating characteristic (ROC) curves showed that sc-Met performs better than S100-β and LDH in the identification of UM liver metastases. Serial measurement in patients with metastatic disease showed a progressive rise of sc-Met levels whereas in disease-free patients, sc-Met levels were stable [330]. None of these biomarkers have entered into clinical practice so far. Indeed, prospective studies with a large cohort of patients are still necessary to identify biomarkers or a combination of them with a predictive value of the early development of metastases.

### Circulating tumor cells, DNA, miRNA, and exosomes

Circulating tumor cells (CTCs) in the blood have been proposed as a prognostic biomarker, potentially useful to monitor the response to therapy in patients with metastatic carcinoma [331]. Since UM metastasizes through hematogenous dissemination, it is conceivable that detection of circulating UM cells into the blood may be useful for monitoring disease progression or response to treatment.

Different technical approaches have been used to detect or isolate UM CTCs, including immunomagnetic sorting [332], enrichment by size of tumor cells [333], or RT-PCR detection of tyrosinase and MelanA/MART1 mRNA [334]. Variable results have been reported on the prognostic value of CTC detection in UM, and comparisons among studies are difficult due to the different methodologies applied.

CTCs expressing the melanoma-associated chondroitin sulfate proteoglycan (MCSP) were isolated by immunomagnetic cell sorting in 10 of 52 patients with primary UM. The presence of CTCs showed correlation with clinical parameters of progression but not with the outcome due to the short follow-up [335]. However, a subsequent study involving 81 primary UM patients did not confirm these results. Indeed, the presence and number of CTCs isolated by the same technique showed no significant association with prognostic parameters or with metastatic progression and no therapy-related changes [336]. A recent study reported the isolation of CTCs by immunomagnetic sorting of CD146 (MUC18)-positive cells, which were further characterized for high molecular weight melanoma-associated antigen (MEL) expression in four of eight patients with primary, not metastatic UM whereas no CTCs were found in four choroidal nevus patient [337]. The small patient sample did not allow a correlation of CTC number and prognosis. Also, the combination of two monoclonal antibodies against a melanoma-associated glycoprotein was used for CTC immunomagnetic sorting from the blood of 31 UM patients without metastatic disease. The presence or number of CTCs did not show a significant association with clinical prognostic factors [332]. One recent study combined the isolation of CTCs with the same technology with immuno-FISH detection of monosomy of chromosome 3 in 44 primary UM patients. Monosomy 3 was detected in intact CTCs of 58% of patients and was confirmed in the primary tumor of 10 out of 11 tested patients [338]. These results suggest that the FISH–cytogenetics of CTCs might provide useful prognostic information, particularly in UM patients not undergoing surgery or biopsy.

A different approach for identifying CTCs is based on RT-PCR analysis of melanoma-associated transcripts. The presence of CTCs detected by RT-PCR of tyrosinase or MelanA/MART1 transcripts represented an independent prognostic factor for metastases and survival, in patients with high-risk primary UM [339]. Also, in metastatic UM patients, the RT-PCR detection of CTCs resulted to be a poor prognostic factor [340].

Therefore, the prognostic value of the presence or number of CTCs in the blood of UM patients differs depending on the methods of detection and further studies are needed to better define the significance of CTCs as a biomarker in UM.

Circulating tumor DNA (ctDNA) has also been detected in different tumors and correlated with disease burden, prognosis, and response to therapy [341]. Most of the circulating free DNA (cfDNA) present in healthy individuals derives from physiologic apoptosis of hematopoietic cells. The contribution of tumor cells on the blood DNA is limited and depends on tumor burden or cell turnover [342]. Therefore, quantification of ctDNA needs to be associated with detection of a tumor-specific mutation that identifies cfDNA of tumor origin. The development of powerful sequencing techniques has improved the limit of detection of ctDNA copies in the total normal DNA from 10% by Sanger sequencing up to 1–2% by ultradeep sequencing [343] and 0.01% by droplet digital PCR (ddPCR) [344]. The comparative analysis of CTC detection and that of the quantification of ctDNA bearing UM-specific GNAQ/GNA11 mutations showed that both CTC and ctDNA levels were associated with the presence of miliary hepatic metastasis and with poor disease-free and overall survival. However, in multivariate analyses, ctDNA was a better prognostic marker than CTCs [345]. A clinical trial (NCT02849145) is recruiting participants to evaluate the ctDNA in the blood, before and after resection of hepatic metastasis of UM and during post-surgery follow-up.

Serum microRNA (miRNA) are also attractive biomarkers of tumor progression and response to therapy and are mostly encapsulated within vesicles of endosomal origin from 30 to 150 nm known as exosomes [346, 347] MicroRNA are small non-coding RNA involved in different biological processes through the regulation of gene expression at the post-transcriptional level. Two studies [348, 349] reported higher levels of miR146a in the serum of patients with primary UM than those of healthy controls, and levels were even higher in patients undergoing metastatic progression. MicroR146a is involved in NK cell function and inflammation and is upregulated by the MAPK cascade, which is activated by GNAQ/GNA11 mutations in UM. A study investigated the presence of exosomes and their miRNA content in the liver perfusate of 12 patients with UM liver metastases. MelanA-positive exosomes were present in the liver circulation in metastatic UM, with similar miRNA profiles among patients. Also, the peripheral blood contained higher levels of exosomes compared to healthy controls [350]. Further studies with the larger number of patients are required to evaluate the role of circulating miRNA and exosomes as biomarkers for the early detection of distant metastases.

## Animal models

A spontaneous UM might be the ideal animal model, provided that it would reproduce the human disease. Although rare cases of spontaneous intraocular melanomas have been reported in different animal species, including dogs [351, 352], cats [353, 354], horses [355], and cattle [356], no spontaneous UMs were reported in mice, which would represent the most convenient species. Syngeneic transplantable and transgenic models of UM allow investigating the interaction between the host immune system and the tumor. On the other hand, *in vivo* xenotransplantation models of human UM require immune-deficient hosts. The subcutaneous implant of patient-derived UM fragments (patient-derived xenografts, PDXs) is of special interest since PDXs reproduce the genetic, histopathological, and biological properties of the original human tumor.

Considering the implant of UM cell suspensions, the injection site is of crucial importance. UM cells may be orthotopically injected in the posterior or anterior chamber of the eye to better mimic primary tumor growth. Different routes of injection may be used to mimic metastatic dissemination, such as injection into the spleen, liver, tail vein, or heart. In addition, the genetic features of human UM cell lines, such as GNAQ or GNA11 mutations, chromosome 3 monosomy, BAP1 mutation, and liver tropism, should be considered.

Hence, the development of UM *in vivo* models of spontaneous liver metastases should be an important issue and may be helpful in the preclinical testing of new therapeutic strategies.

### Syngeneic models

Most syngeneic models were based on the mouse cutaneous melanoma cell line B16 or its metastatic variant B16F10 [357, 358], mostly injected into the eye of C57BL6 mice. In addition, the subline B16LS9 has been derived by repeated *in vivo* passages to select for melanoma cells with metastatic tropism to the liver, to better reproduce the human metastatic disease. Intraocular injection of B16LS9 cells has been utilized as an orthotopic syngeneic model producing liver metastases [359]. The syngeneic orthotopic models have been used to study immunologic aspects of UM development such as the inhibitory effect of NK T cell-derived IL-10 on the anti-metastatic activity of NK cells [360]. A recent study showed that activation of NK cells by administration of the Toll-like receptor-5 agonist entolimod suppressed the development of spontaneous hepatic metastases in the orthotopic B16LS9 model [361]. The growth of primary ocular tumor derived by the orthotopic implant of B16LS9 cells with the p53 activator Nutlin-3 and the topoisomerase inhibitor topotecan delayed tumor growth [362]. Although relevant information on UM derives from syngeneic models, it should be taken into account that the results obtained using B16 melanoma cells or their derivatives may not reflect the biology of the human UM. B16 cells carry a BRAF mutation that is very rare in UM.

### Xenotransplantation models

Historically, the first xenotransplantation animal models mimicking UM were developed by implantation of hamster melanoma tissue in the anterior chamber of rabbits (Greene HS PMID: 5934903). Human xenograft models of UM have been established in immune-deficient mice [363] and rats [364], rabbits [365], and zebrafish [366]. Established primary uveal melanoma cell lines have been injected into the anterior or posterior chamber and into the ciliary body or choroid. For example, OCM1, OCM3, OCM8, EOM3, 92.1, OM431, and MEL202 were injected intracamerally into nu/nu BALB/c (H-2d) mice [367]. In this study, OCM1 and 92.1 cells gave rise to liver metastasis at day 50 with an incidence of 80%. However, the OCM1 cell line harbors the BRAF V600E mutation typical of CM, while it is wild type for GNAQ, GNA11, and BAP1. Differently, 92.1 cell bears the GNAQ Q209L driver mutation, but is wild type for the BAP1 gene. In addition, both cell lines are disomic for chromosome 3 and therefore lack the main features of UM at high metastatic risk.

Patients with ciliary body or choroid melanomas have metastasis with higher frequency and have a worse prognosis. To mimic this situation, MEL290 UM cells, transduced by lentiviral EGFP encoding for a fluorescent protein, were injected into the posterior chamber of the eye of nu/nu mice. This approach led to micrometastasis development into the liver at 4–6 weeks after transplantation with an incidence of 100% [368]. Moreover, more liver micrometastases developed in the intraocular model than in the tail injection model.

Orthotopic xenograft models have been used for the preclinical assessment of new treatments [369–371]. For example, the UM cell line OMM1.3, bearing a GNAQ Q209P mutation, retro-orbitally injected into nu/nu mice developed liver metastases 9 weeks post injection. In this model, treatment with the c-Met/ALK tyrosine kinase inhibitor crizotinib prevented liver metastasis development without blocking ocular tumor growth [98]. These data point out to an important role of the HGF/c-Met axis in metastatic spreading of UM to the liver.

The majority of human metastatic UM has chromosome 3 loss and BAP1 mutation; however, xenograft models using monosomic human UM cells have not been developed so far. Surprisingly, OCM1A, 92.1, and Mel290 cell lines, silenced for the expression of BAP1, gave rise to smaller tumors or developed fewer metastases than BAP1 WT cells when injected subcutaneously or intravenously, respectively [209].

Pseudo-metastatic xenotransplantation models of UM have also been obtained by intracardiac or tail vein injection [368, 372] or by implant into the liver [325, 373] or spleen [89, 374, 375]. Spleen transplantation allows UM cells to reach and colonize the liver through the portal circulation. It has been used as a *pseudo-metastatic model* to evaluate treatment, mechanisms of metastasis, or UM biomarkers [89, 330, 376–378]. For example, preliminary detection of human-soluble sc-Met in the serum of UM xenograft led to the identification of this molecule as a potential biomarker of metastases in UM patients [330]. The human GNAQ-mutated UM cell line TJU-UM001, transplanted under the spleen capsule of NSG mice, developed multiple hepatic metastases whereas lung metastases were not observed. Interestingly, a laminin-rich vasculogenic mimicry pattern was seen in the hepatic lesions similar to that observed in primary and metastatic human UMs [379].

PDXs were generated from primary or metastatic UM tissues to propagate tumors *in vivo*, for pathogenesis and preclinical therapy studies. Transplantation of UM fragments into the subcutis or the interscapular fat pad of nu/nu [380] or SCID [381] mice yielded a low take rate. Tumor takes were significantly higher when implanted fragments were derived from metastases as compared to those derived from primary tumors (52.9%). Interestingly, engraftment of metastatic tumors was predictive of a short overall survival of metastatic patients. In addition, xenograft growth of primary tumors inversely correlated with 5-year metastasis-free survival [381]. The comparison of serial UM xenografts to the original tumor showed that xenografts are genetically stable over *in vivo* passages and maintain the genetics features of the original human UM [382]. Despite the limits of a low rate of engraftment and growth delay after initial transplantation, panels of UM PDXs have been employed to test combinations of targeted therapies [383, 384].

Human UM cell line xenografts have also been developed in rabbits which bear larger eyes than mice and are therefore more suitable for examination and surgical procedures, upon immunosuppression with cyclosporine-A. However, in these models, most of the metastases were found in the lungs and rarely in the liver [385–387].

Injection of human UM cell lines into the chick embryo, which lacks an immune system till days 10–15, has also been used as an easily accessible and economical xenograft model [388, 222]. Preliminary data of the chick embryo metastasis model show that OMM1 and 92.1 cells injected into the circulation of 7-day embryos mostly colonize tissues of neural crest derivation [389].

Recently, human UM xenotransplantation models were established in zebrafish using cell lines derived from primary UM or metastatic UM expressing a red fluorescent protein. This model was suitable to test the efficacy of different experimental anticancer drugs (e.g., quisinostat and the neddylation [ubiquitin-like protein NEDD8 conjugation to target proteins] pathway inhibitor MLN-4924) which repressed the migration and proliferation of UM cells. As the drugs were dissolved in the water, the rate of drug absorbance into zebrafish embryos needs a better characterization [390].

### Transgenic mouse models

Transgenic animals, which constitutively express an oncogene, represent a useful tool for the study of tumorigenesis *in vivo* and to explore tumor–host interactions in the tumor microenvironment [391]. Several transgenic mouse models of CM, which resemble the biological features of the human disease, also develop a choroidal melanoma, although they do not bear mutations typical of human UM [265]. Very recent studies exploited the activated GNAQ oncogene, which is found in a significant fraction of human UM, to develop transgenic UM models.

One of the earliest CM models has been the MT-ret transgenic mice, which express the human RET oncogene controlled by the mouse metallothionein I promoter–enhancer. These mice develop hyperplastic and tumoral lesions in different melanocyte-containing tissues [392]. Although Ret kinase mutations have not been found in human melanomas, the RET oncogene stimulates the downstream MAP kinase pathways, which are typical of melanoma [393] and may explain melanoma formation, in the transgenic mice. These mice also develop UM as an early event, revealed by exophthalmos [392]. MT-ret transgenic mice were also crossed with ADD transgenic mice, which express the chimeric MHC molecule AAD (HLA-A2/H2-Dd). These MT-ret/AAD mice are suitable for analyzing CD8+ T cell responses directed against immunogenic antigens found in HLA-A2+ melanoma patients [394]. This model allowed establishing the pro-metastatic effects of macrophages, which could be abrogated by treatment with the CSF-1R-specific kinase inhibitor Ki20227 [266]. In another study, the effect of the pro-inflammatory and autoimmune background of the non-obese diabetic (NOD) strain was assessed by back-crossing MT-ret with NOD mice, which resulted in rapid UM development. Dectin-1 expression was reduced in granulocytic myeloid cells in relationship with an increase of regulatory T cells and that of IFN-γ-producing CD8+ T cells in tumors. IFN-γ-inducible nitric oxide synthase (Nos2) was involved in inflammation and accelerated tumorigenesis. These data point to a role of inflammation in UM development and to dectin-1 and Nos2 as potential therapeutic targets [395].

Mice bearing targeted deletions of oncosuppressor genes can be generated to mimic a cancer-prone condition. These mice can be eventually crossed with transgenic mice bearing active oncogenes to enhance the oncogenic effect of the transgene. This is the case of spontaneous malignant melanomas occurring in male Tyr-RAS+ Ink4a/Arf−/− transgenic mice. In these mice, a human tyrosinase enhancer/promoter element drives melanocyte-specific expression of a human activated H-ras gene integrated into the Y chromosome. Such transgenic mice crossed in an Ink4a/Arf-deficient background (Tyr-RAS+ Ink4a/Arf−/−) develop CM and UM, in males [396]. These UMs showed epithelioid- and spindle-shaped features and local invasiveness resembling the human counterpart but no metastatic progression [397].

In Tg(Grm1)EPv transgenic mice, the metabotropic glutamate receptor 1 (Grm1) gene is under the control of the dopachrome tautomerase (Dct, Trp2) promoter, specifically expressed in melanocytes [398]. These mice develop melanomas, in the hairless skin of ear, tail, and anus, and also develop choroidal hyperplastic lesions and UM-like tumors. Furthermore, GRM1 is expressed in human uveal and skin melanoma, suggesting that the glutamate signaling pathway is a possible target for UM therapy [399].

Recently, the first transgenic mouse model, reproducing genetic defects of human UM, has been developed. A transgenic mouse strain, expressing oncogenic GNAQ(Q209L) under control of the Rosa26 promoter, showed not only UM but also dermal nevi and other melanocytic tumors of the central nervous system [400]. In this model, the Yap protein was activated in the eyes, and uveal blood vessels showed melanocytic invasion. However, most of these mice developed metastases only in the lungs. This model is the first transgenic mouse model using a relevant oncogene of human UM and may represent a tool for testing new targeted therapies *in vivo*.

A transgenic model of spontaneous UM was also established in zebrafish, engineered to express oncogenic GNAQ(Q209P) in the melanocyte lineage. These zebrafishes display hyperplasia of uveal melanocytes, but no evidence of tumor progression. However, the combined expression of GNAQ(Q209P) with p53 oncosuppressor inactivation mediated an earlier onset of hyperplastic lesions that evolved in UM. A weak phosphorylation of ERK1/2 was observed in transgenic UM, while staining for nuclear YAP was evident. Therefore, enhanced ERK1/2 signaling seems not a major feature of GNAQ(Q209P)-driven transgenic UM [401].

Recently, cells from a genetically modified mouse strain were used to identify new potential targets for UM. Ric-8A is a molecular chaperone that folds GNAQ and GNA11 subunits and may therefore serve as a target to reduce the abundance of these oncoproteins in UM. Melanocytes obtained from a conditional Ric-8AFlox/Flox; Rosa-CreER+/− mouse strain transduced with the human GNAQQ209L showed autonomous cell proliferation *in vitro* and, upon grafting in mice, developed melanoma tumors. Deletion of Ric-8A in GNAQ Q209L-transduced cells by tamoxifen reduced Gαq-Q209L levels and prevented tumorigenesis *in vivo*. Also, phorbol ester (TPA) treatment inhibited GNAQ Q209L cell proliferation *in vitro* and *in vivo*. The authors propose Ric-8A inhibition or phorbol esters as novel potential treatments of UM [402].

## Conclusions

The last years have brought a dramatic progress of our understanding of UM. The evolution of this rare cancer is now described in considerable detail in terms of cytogenetic, genetic, and transcriptional alterations as well as the signaling pathways involved in the formation of UM and its progression to metastasis. We still have a limited understanding of several central aspects of the disease:

- The etiology of UM
- The ontogenetic cellular origin of UM and its precursor cells
- The role in metastasis of chromosome 3 monosomy as opposed to losses of single regions of the same chromosome
- The molecular basis of the effects of minor cytogenetic alterations (6p, 1p)
- The precise way of action of BAP1
- Metastatic players in BAP1/SF3B1 wild-type metastatic UM
- The role of epigenetic alterations
- The determinants of the liver tropism of UM metastases.

Most importantly, our understanding has so far not led to a sensible improvement in survival of patients with metastatic UM. Research should therefore be focused on the translation of the mechanistic understanding of the disease into proposals for clinical trials using drugs that are targeted at UM-specific molecular alterations. This should hopefully lead to an increase in survival of metastatic patients similar to what has recently been observed for CM patients.

## Footnotes

1
ClinicalTrials.gov identifiers are indicated in parentheses for clinical trials throughout the text.
